# Self-Fluxing Mechanism in Geopolymerization for Low-Sintering Temperature of Ceramic

**DOI:** 10.3390/ma14061325

**Published:** 2021-03-10

**Authors:** Noorina Hidayu Jamil, Mohd. Mustafa Al Bakri Abdullah, Faizul Che Pa, Hasmaliza Mohamad, Wan Mohd Arif W. Ibrahim, Penphitcha Amonpattaratkit, Joanna Gondro, Wojciech Sochacki, Norfadhilah Ibrahim

**Affiliations:** 1 Geopolymer & Green Technology, Centre of Excellence (CEGeoGTech), Universiti Malaysia Perlis (UniMAP), Kangar 01000, Perlis, Malaysia; faizul@unimap.edu.my; 2 Faculty of Mechanical Engineering Technology, Universiti Malaysia Perlis (UniMAP), Kangar 01000, Perlis, Malaysia; 3 Faculty of Chemical Engineering Technology, Universiti Malaysia Perlis (UniMAP), Kangar 01000, Perlis, Malaysia; wmarif@unimap.edu.my; 4 Biomaterial Research Niche Group, School of Materials and Mineral Resources Engineering, Universiti Sains Malaysia, Nibong Tebal 14300, Penang, Malaysia; hasmaliza@usm.my; 5 Synchrotron Light Research Institute, 111 University Avenue, Muang District, Nakhon Ratchasima 30000, Thailand; penphitcha@slri.or.th; 6 Department of Physics, Częstochowa University of Technology, 42-200 Częstochowa, Poland; joanna.gondro@pcz.pl; 7 Faculty of Mechanical Engineering and Computer Science, Częstochowa University of Technology, 42-200 Częstochowa, Poland; w.sochacki@imipkm.pcz.pl; 8 Faculty of Bioengineering and Technology, Universiti Malaysia Kelantan (Jeli Campus), Jeli 17600, Kelantan Darul Naim, Malaysia; nfadhilah@umk.edu.my

**Keywords:** ceramic, geopolymer, self-fluxing, sintering, kaolin, sintered geopolymer

## Abstract

Kaolin, theoretically known as having low reactivity during geopolymerization, was used as a source of aluminosilicate materials in this study. Due to this concern, it is challenging to directly produce kaolin geopolymers without pre-treatment. The addition of ground granulated blast furnace slag (GGBS) accelerated the geopolymerization process. Kaolin–GGBS geopolymer ceramic was prepared at a low sintering temperature due to the reaction of the chemical composition during the initial stage of geopolymerization. The objective of this work was to study the influence of the chemical composition towards sintering temperature of sintered kaolin–GGBS geopolymer. Kaolin–GGBS geopolymer was prepared with a ratio of solid to liquid 2:1 and cured at 60 °C for 14 days. The cured geopolymer was sintered at different temperatures: 800, 900, 1000, and 1100 °C. Sintering at 900 °C resulted in the highest compressive strength due to the formation of densified microstructure, while higher sintering temperature led to the formation of interconnected pores. The difference in the X-ray absorption near edge structure (XANES) spectra was related to the phases obtained from the X-ray diffraction analysis, such as akermanite and anothite. Thermal analysis indicated the stability of sintered kaolin–GGBS geopolymer when exposed to 1100 °C, proving that kaolin can be directly used without heat treatment in geopolymers. The geopolymerization process facilitates the stability of cured samples when directly sintered, as well as plays a significant role as a self-fluxing agent to reduce the sintering temperature when producing sintered kaolin–GGBS geopolymers.

## 1. Introduction

Geopolymer products have become increasingly important in industry because of their ecological safety, economic efficiency, and diverse applications [1,2,3,4]. Geopolymers have found application in virtually all fields of industry, providing high mechanical strength, high chemical inertness, and excellent fire resistance. They are considered a replacement for conventional cement-based components as well as for ceramic parts that can be used in medium–high temperatures typically below 1200 °C [5,6]. Geopolymers are produced by a geosynthesis process involving natural or synthetic aluminosilicates in which amorphous silicon and aluminum oxide react in a strongly basic medium to form networks which are chemically and structurally like natural rock [7,8].

The geopolymerization process is described by the following stages: under high alkaline condition, dissolving of oxide minerals from the alumina–silica-rich source materials; transportation/orientation of dissolved oxide minerals followed by gelation; and polycondensation to develop a 3D stable network of silicoaluminate structures [9]. The geopolymerization process depends on many parameters, including the chemical and mineralogical composition of the starting materials, curing temperature, water content, and concentration of the alkaline compound [10]. Raw kaolin has been considered for the synthesis of geopolymers without preliminary thermal treatment to limit the overall energy demand for calcination to improve the sustainability of the process [11,12]. Calcium-rich aluminosilicates, such as ground granulated blast furnace slag (GGBS), are sometimes used with low reactive precursors, such as class F fly ash (FA), to boost the reactivity of the system, which eliminates the need for heat curing [9]. The use of raw kaolin in geopolymer was considered in a previous work [13].

Sintering of geopolymers always leads to the formation of sintered ceramic bodies with improved properties [14]. Ceramic products containing naturally occurring rocks and minerals as starting materials must undergo a special process to control the purity, particle size, particle size distribution, and heterogeneity. These factors play a significant role in the final properties of the finished ceramic [15]. The reaction sintering method is influenced by the impurities and mineralogical structure of kaolin minerals [16]. The most common methods of forming ceramics include extrusion, slip casting, pressing, tape casting, and injection molding. Then, these green ceramics undergo heat treatment to produce a rigid, finished product [17]. Conventional approaches of sintering glass ceramics usually include two steps: vitrifying raw materials at high temperatures (1300–1500 °C), followed by nucleation and crystal growth [18].

Low-sintered ceramics have attracted many researchers in the last few decades. Several techniques of lowering ceramic temperature, requiring a complicated setup and high manufacturing cost, have been developed. Geopolymerization has become an attractive research topic in ceramic sintering due to its low energy consumption during the fabrication process. The objective of this research was to distinguish the contribution of the chemical composition from kaolin, GGBS, sodium silicate, and sodium hydroxide to the phase transformation of sintered kaolin–GGBS geopolymers. These will lead to the understanding of the chemical composition, which act as a self-fluxing agent during geopolymerization and will helped researchers to design low-sintered ceramic.

## 2. Materials and Methods

The geopolymer samples produced from kaolin and GGBS were mixed with an alkali activator (Ann Joo Integrated Steel Sdn. Bhd., Penang, Malaysia) using a mechanical stirrer with a solid-to-liquid ratio of 2:1 until slurry. The chemical compositions of kaolin and GGBS obtained from the X-ray fluorescence characterization are shown in Table 1. Both reported its main chemical composition of aluminosilicate source, which are SiO_2_ and Al_2_O_3_.

**Figure 1:**
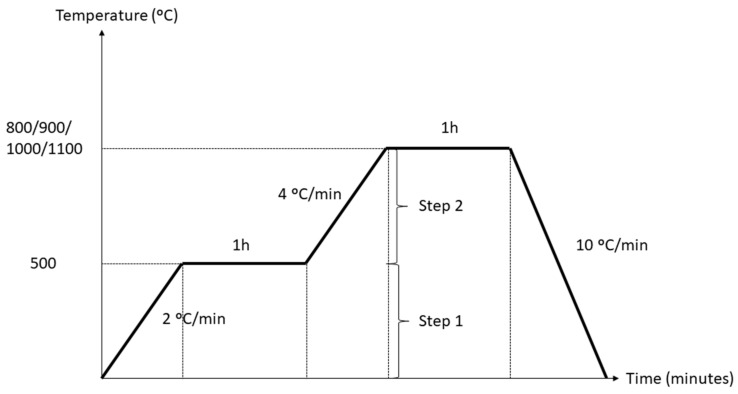
Two-steps of sintering profile for sintering kaolin–GGBS geopolymer.

The homogenized mixture was poured into a mold. The alkali activator was prepared 24 h prior to use by mixing 8 M NaOH (Formosa Plastic Corporation, Taiwan) and sodium silicate (South Pacific Chemical Industries Sdn. Bhd., Petaling Jaya, Malaysia) with Na_2_SiO_3_ to NaOH ratio of 4:1. The slurry geopolymer was vibrated to remove trapped air and sealed with plastic at the exposed portion of the mold during the curing stage. Samples cured at room temperature for 24 h, followed by curing at 60 °C in the oven for 14 days. The cured samples were sintered with the heating profile shown in Figure 1. Step 1 was sintered to a lower temperature, 500 °C, with a heating rate of 2 °C/min. Higher temperature settings were applied in Step 2, ranging from 800, 900, 1000, and 1100 °C, with a heating rate of 4 °C/min. The soaking time was set to 1 h for each step. The cooling rate was 10 °C/min. The kaolin–GGBS geopolymer was sintered using a bench top muffle furnace. Two steps of sintering were applied in this method to purposely control the major cracking effect on the sintered samples.

The phase composition of the raw materials and kaolin–GGBS geopolymer was determined using the X-ray diffraction method (Bruker D8 Advance, USA), equipped with a copper anode (Cu Ka, λ 1.5406 Å). Prior to the analysis, the dry powder was compacted, and the analysis was recorded within a 2θ range of 5–80 °C, with a scan rate of 0.1 deg/s. Next, the data were analyzed using X’Pert HighScore Plus software. The image of the microstructure was captured using a scanning electron microscopy (ZeissSupra 35VP, Jerman), which worked at an accelerating voltage of 5 kV. The specimens were cut into small pieces with the edges ground down to make them smooth. The samples were vacuumed for an hour and the surfaces were coated with Au–Pd for imaging purposes.

The micro-X-ray absorption spectroscopy experiments were conducted at BL8 beamline at the Synchrotron Light Research Institute (SLRI), Thailand. The X-ray absorption near edge structure (XANES) of calcium atoms in the four different sintering temperatures of sintered kaolin–GGBS geopolymers samples were measured. One sample of kaolin–GGBS geopolymer was characterized for determination of changes in calcium atoms after sintering. The photon energy of the synchrotron X-rays was scanned by a Ge(220) double-crystal monochromator to excite the samples in the K-edge XANES regions of calcium. The X-ray absorption was determined by I_f_/I_0_ where I_0_ is the incident photon intensity I_f_ is the fluorescent signal. The X-ray absorption is given by ln(I_0_/I_t_), where It is the transmitted photon intensity after the standard. The photon energy was calibrated against the K edge of copper foil at 8979 ± 0.3 eV. With use of the graphical utilities ATHENA in IFEFFIT all XANES spectra were normalized to atomic background absorption in the post-edge region. The theoretical XANES signals were simulated using the FEFF8.2 code.

## 3. Results

### 3.1. Compressive Strength

**Figure 2:**
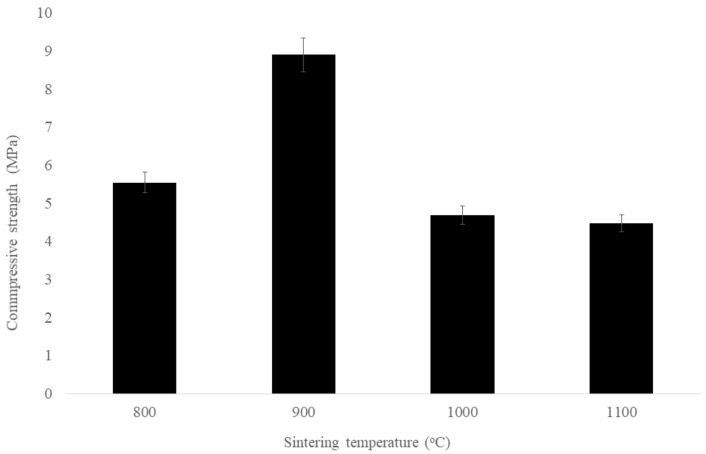
Effect of sintering temperature on the compressive strength of sintered kaolin-GGBS geopolymer.

Figure 2 shows the compressive strength of sintered kaolin–GGBS geopolymer at different sintering temperatures. The heating rate, sintering temperature of T1, and soaking time were kept constant while the sintering temperature of T2 was 800, 900, 1000, or 1100 °C. The compressive strength firstly increased and then decreased with the increase in sintering temperature, reaching a maximal value of 9 MPa at 900 °C. Previous research reported the compressive strength of kaolin geopolymer after 24 h curing in 60 °C. However, the strength obtained was only 1 MPa [19].

We found that sintering at a temperature lower than 1000 °C produces sintered kaolin-GGBS geopolymer with high compressive strength. The major influencing factor is the pore distribution of the sintered kaolin–GGBS geopolymer. It implies that a higher temperature drastically changes the microstructure. The second influencing factor is the crystalline phase, which determines the strength of glass ceramic. A high volume fraction of crystallinity should be achieved to obtain high compressive strength.

### 3.2. Phase Transformation Analysis

**Figure 3:**
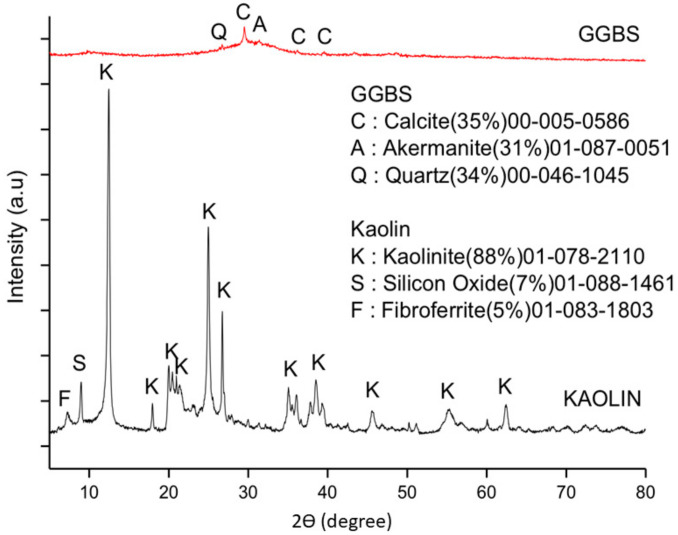
Phase composition of kaolin and ground granulated blast furnace slag (GGBS).

The diffraction patterns of raw kaolin and GGBS are shown in Figure 3. Based on the semi-quantitative analysis, the major phase composition of kaolin was 88% kaolinite (Al_4_(OH)_8_(Si_4_O_10_)) (00-005-0586). The minor phases was 7% silicon oxide (SiO_2_)(01-088-1461), and 5% fibroferrite (Fe(OH)(SO_4_)(H_2_O)_5_) (01-083-1803) in the crystalline phases. The GGBS consisted of calcite (CaCO_3_) (00-005-0586) ~35%, akermanite (Ca_2_Mg(Si_2_O_7_) (01-087-0051) ~32%, and quartz (SiO_2_) (00-046-1045) ~34%. The phase quantification of semi-quantitative analysis using Xpert HighScore Plus software was only conducted for the crystalline phase.

**Figure 4:**
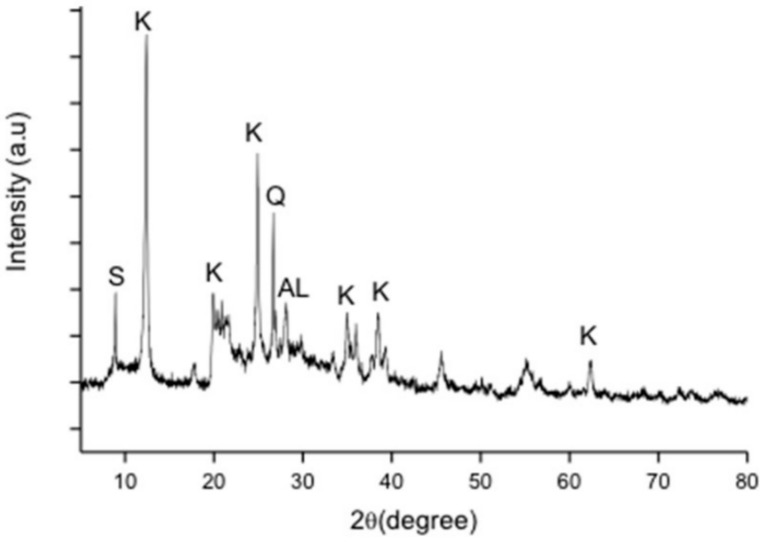
Phase composition of kaolin–GGBS geopolymer after curing for 14 days. (S: Silicon Oxide, K: Kaolinite, Q: Quartz, AL: Albite).

Figure 4 shows the phase composition of kaolin–GGBS geopolymer after curing for 14 days in an oven at 60 °C. There were significant differences in the phase composition after curing compared with the composition in raw kaolin and GGBS. The crystalline phase of kaolinite (Al_4_(OH)_8_(Si_4_O_10_)) and the silicon oxide (SiO_2_) phase from kaolin remained in the kaolin–GGBS geopolymer after the curing phase. The quartz (SiO_2_) phase from GGBS was observed in the as-cured samples. However, a new phase was observed as albite Na(AlSi_3_O_8_), indicating the reaction of kaolin–GGBS and the alkali activator during geopolymerization. The phase was drastically transformed after sintering as shown in all sintered kaolin–GGBS geopolymer.

**Figure 5:**
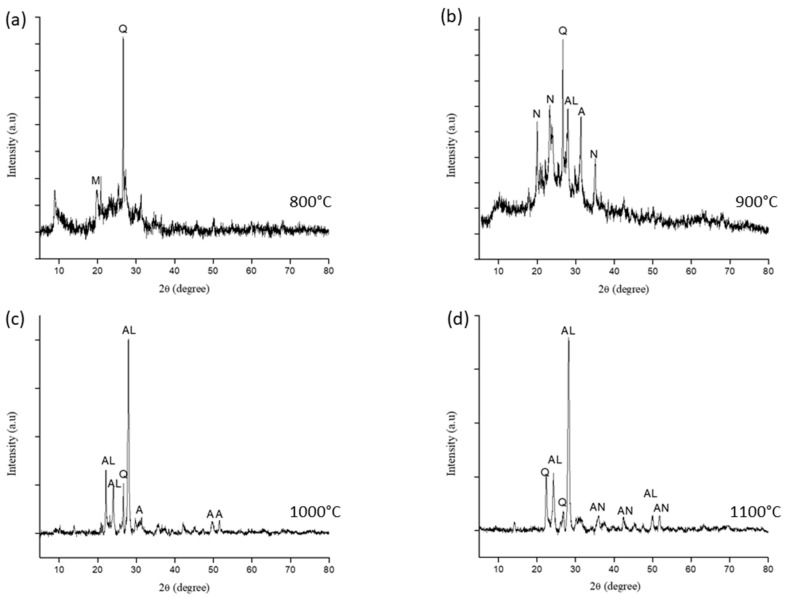
The phase transformation of sintered kaolin–GGBS geopolymer at different sintering temperatures (**a**) 800 °C, (**b**) 900 °C, (**c**) 1000 °C, and (**d**) 1100 °C.

Figure 5 shows the phase transformation of the sintered kaolin–GGBS geopolymer at different sintering temperatures, and percentages of the phase composition are presented in Table 2. Sintering at 800 °C resulted in the formation of the magnetite and quartz phase. The existence of magnetite was related to the deformation of the fibroferrite phase from raw kaolin during sintering. The intensity of quartz increased after the sintering processes at 800 and 900 °C. The semi-quantitative analysis indicated a 77% composition of quartz in 800 °C sintered kaolin–GGBS geopolymer, which could be attributed to the high compressive strength compared with samples sintered at 1000 and 1100 °C. This is due to the known properties of quartz, which has high compressive strength [20].

The composition of quartz as decreased to 4% in 900 °C sintered sample, although the compressive strength obtained was the highest at this temperature. This was due to the formation of 10% akermanite and 13% nepheline. Akermanite (Ca_2_MgSi_2_O_7_) has recently received interest among researchers due to its mechanical properties, which are better than those of hydroxyapatite (HA) [18]. It has a typical degradation rate compared to other silicate bioceramics [20]. Akermanite belongs to the melilite group, which has excellent wear and corrosion resistance [21].

The peak formations at 1000 and 1100 °C demonstrated crystalline peaks at the positions of 2Ɵ, 20 to 30 degrees. The intensity of the akermanite peak was compared between the sintering temperature of 900 and 1000 °C. However, the albite phase showed a highly crystalline process in 1000 °C sintered kaolin–GGBS geopolymer. Albite occurred due to the transformation of nepheline when excessive Al_2_O_3_ and Na_2_O were present during sintering at 1000 °C. The value of viscosity during sintering and crystallization was significant. The lesser the viscosity value, the more rapidly the crystallization process occurs. This can hinder sintering and create a large number of porosities. However, if the viscosity value is too high, the crystallization process will be time-consuming.

The presence of the anorthite phase at 1000 °C sintered samples showed that the primary calcite phase from the GGBS decomposed during sintering due to the heating process above the decarbonization temperature. CaO, which originates from calcite and akermanite, aiding the formation of the anorthite phase within an adequate time and appropriate temperature for the reactions to form Ca silicates, which is anorthite. It can be seen from the XRD patterns that the peak quartz (SiO_2_) and akermanite (Ca_2_Mg(Si_2_O_7_) phase decreased at 1000 °C. The released SiO_2_ and CaO from these phases can be used to initiate the formation of the anorthite phase. The results implied that the kaolinite gives plasticity to the ceramic mixture; thus, quartz (SiO_2_) maintains the shape of the formed product during sintering, and Na_2_O from the alkali activator serves as a self-fluxing agent.

The phase transformation of sintered kaolin–GGBS geopolymer with different sintering temperatures is expressed in the form of chemical reactions, shown in Equations (1)–(4). However, the equations are not precisely balanced for many ceramic products are relatively non-stoichiometric. Furthermore, the various oxides in the starting powder can induce a liquid phase during sintering. The presence of a liquid phase can slightly shift the formation temperature of each phase and its amount.

According to Liu et al. (2019), the structure of GGBS with a high content of the amorphous phase contains some calcium ions coated by silicate network, for example, SiO_4_ tetrahedron or the –Si–O–Ca–O–Si– structure. Therefore, the silicate network is regarded as a protective film on the surface of slag particles to avoid corrosion by water. The OH^–^ ions can accelerate the corrosion of protective film and the network structure collapse by moving toward the core of GGBS particles. The Ca^2+^ ions and SiO_4_^2−^ ions detach from the silicate network. The akermanite phase generated in interstitial solution densifies the area. This enhances the strength of sintered kaolin–GGBS geopolymer [21].

Sintering at 800 °C:

SiO_2_→ SiO_2_ (Amorphous)(Crystalline) (1)

Sintering at 900 °C:

(2) $$\begin{array}{cccc} {\mathrm{Na}}_{2}\mathrm{Si}O_{3} & +\;\mathrm{NaOH} & \to \;{\mathrm{Na}}_{7}({\mathrm{Al}}_{6}{\mathrm{Si}}_{10}O_{32}) & +\;\mathrm{Na}(\mathrm{Al}{\mathrm{Si}}_{3}O_{8})\\ \left(\mathrm{Kaolinite}\right) & \left(\mathrm{Alkali}\;\mathrm{activator}\right) & \left(\mathrm{Nepheline}\right) & \left(\mathrm{Albite}\right) \end{array}$$

Sintering at 1000 °C:

(3) $$\begin{array}{ccccc} {\mathrm{Al}}_{4}{\left(\mathrm{OH}\right)}_{8}({\mathrm{Si}}_{4}O_{10}) & +\;{\mathrm{CaCO}}_{3} & +\;{\mathrm{Na}}_{2}{\mathrm{SiO}}_{3}\;+\;\mathrm{NaOH} & \to \;{\mathrm{Ca}}_{2}\mathrm{Mg}({\mathrm{Si}}_{2}O_{7}) & +\;\mathrm{Na}(\mathrm{Al}{\mathrm{Si}}_{3}O_{8})\\ \left(\mathrm{Kaolinite}\right) & \left(\mathrm{calcite}\right) & \left(\mathrm{Alkali}\;\mathrm{activator}\right) & \left(\mathrm{Akermanite}\right) & \left(\mathrm{Albite}\right) \end{array}$$

Sintering at 1100 °C:

(4) $$\begin{array}{ccccc} {\mathrm{Al}}_{4}{\left(\mathrm{OH}\right)}_{8}({\mathrm{Si}}_{4}O_{10}) & +\;{\mathrm{CaCO}}_{3} & +\;{\mathrm{Na}}_{2}{\mathrm{SiO}}_{3}\;+\;\mathrm{NaOH} & \to \;\mathrm{Ca}{\mathrm{Al}}_{2}{\mathrm{Si}}_{2}O_{8}) & +\;\mathrm{Na}(\mathrm{Al}{\mathrm{Si}}_{3}O_{8})\\ \left(\mathrm{Kaolinite}\right) & \left(\mathrm{calcite}\right) & \left(\mathrm{Alkali}\;\mathrm{activator}\right) & \left(\mathrm{Anorthite}\right) & \left(\mathrm{Albite}\right) \end{array}$$

### 3.3. Microstructural Evolution at Different Sintering Temperature

**Figure 6:**
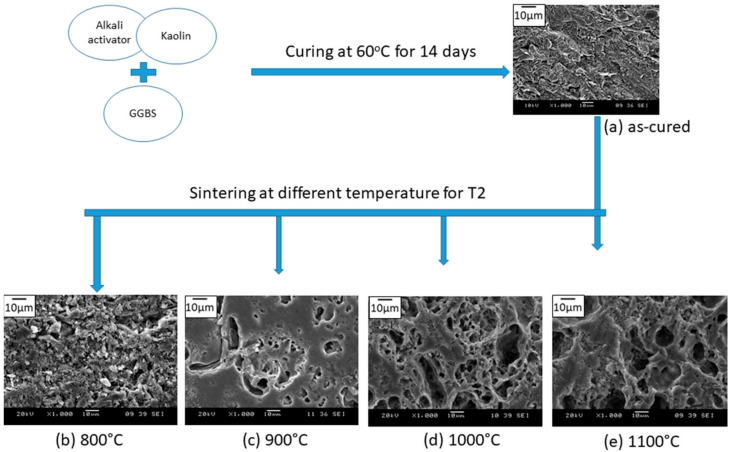
Microstructural evolution of sintered kaolin–GGBS geopolymer at different sintering temperatures with magnification 1000×: (**a**) as-cured, (**b**) 800 °C, (**c**) 900 °C, (**d**) 1000 °C, and (**e**) 1100 °C.

Figure 6 compares the microstructure of sintered kaolin–GGBS geopolymer evolution with the microstructure of the as-cured geopolymer. A similar microstructure can be observed in Figure 6a for as-cured and Figure 6b for sintered at 800 °C. The figures indicate that a low sintering temperature resulted in an incomplete reaction of kaolin–GGBS. Furthermore, it implied an initial stage of densification for the 800 °C sintered kaolin–GGBS geopolymer. The amount and size of pores increased at 1000 and 1100 °C. The sintering processes at 1000 and 1100 °C both showed incomplete densification, with high porosity in the sintered samples. The pores became primarily network-like or interconnected, which led to an increase in porosity. The formation of interconnected pores can be correlated with the drop in compressive strength in these two sintered samples. A spot of granular grain structure was present in the 1100 °C sintered samples.

The densified area was clearly seen in the 900 °C sintered samples. A formation of open pores with irregular shape was obtained from the sintering process. However, more densified areas were observed at 900 °C. These can be related to the contribution of MgO toward densification and pore shape, which led to the high compressive strength for this sample. This can be verified by the akermanite (Ca_2_Mg(Si_2_O_7_) phase, which existed at 900 °C from the X-ray diffraction result. Research conducted by Li et al. proved that the addition of reactive MgO into the geopolymer paste would refine the pore size, increasing the compressive strength. The nepheline and albite, consisting of Na_2_O, functioned as a self-fluxing agent to complete the sintered geopolymerization, with densified properties at low sintering temperatures [22].

### 3.4. Micro-X-ray Adsorption Analysis

**Figure 7:**
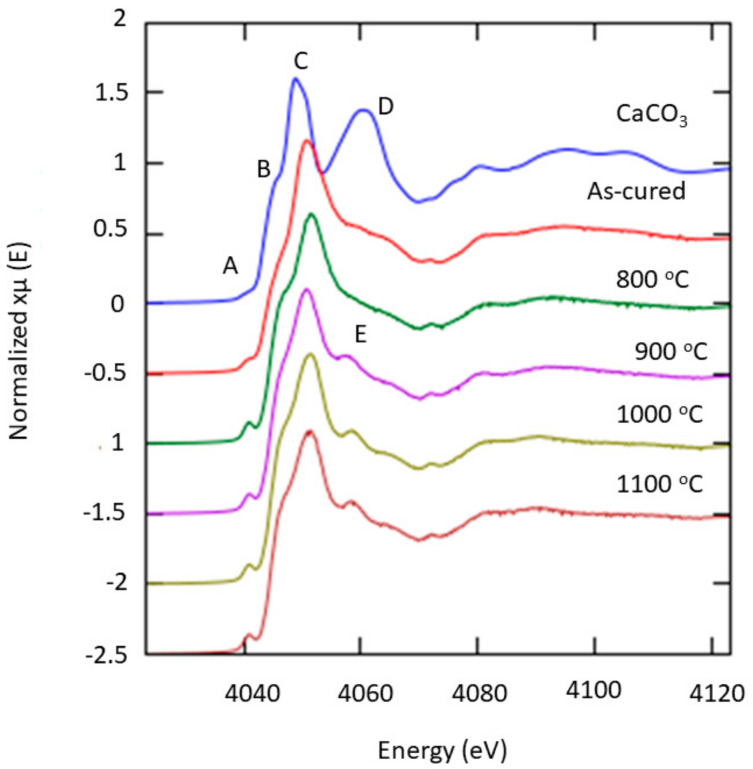
Normalized X-ray absorption near edge structure (XANES) spectra at the Ca K-edge: Comparison between before sintering samples and sintered kaolin–GGBS geopolymer at different temperature.

The Ca K-edge XANES spectra of the sintered kaolin–GGBS geopolymer with a different sintering temperature are presented in Figure 7. The spectra had a great absorption range from 4000 to 4200 eV. The sintered kaolin–GGBS geopolymer spectra were compared with the CaCO_3_ standard. From the XANES analysis, five XANES features, labelled from A to E, were visible as the energy increased. No significant changes were observed in the extended x-ray absorption fine structure, (EXAFS) region. The pre-edge peaks (labelled as A) increased as the sintering temperature increased. Conversely, the near-edge peaks (labelled as B) changed when the samples were sintered. The peak drastically increased compared with the as-cured sample as the sintering temperature increased, except at the 800 °C sintering temperature.

The dominant peak (labelled C) remained unchanged compared to the CaCO_3_ standard. There was no occurrence of the post-edge peak of CaCO_3_ in sintered geopolymer indicated by the peak (labelled as D) in the CaCO_3_ standard. The results showed that a new peak (labelled as E) occurred, which increased when sintering was performed above 800 °C. The first small feature at the pre-edge peak A can be ascribed to the 1s–3d transition, which is commonly observed in the K-edge spectra of the first-row transition metals. Peak B was assigned to the 1s–4s transition. The most intense peak C was due to the 1s–np transition. It was the principal peak that followed the dipole transition selection rules. The shoulder after the main resonance peak D was mostly from multiple scattering processes, which was very sensitive to the immediate surroundings of Ca. The new peak E at 900 °C sintered kaolin–GGBS geopolymer was related to the Ca phase.

The differences in the XANES spectra were related to the phases obtained from the X-ray diffraction analysis and microstructure analysis The X-ray diffraction measurements indicated the presence of akermanite and anorthite at sintering temperatures of 1000 and 1100 °C, respectively, implying that the high intensity of the edge peak at these temperature readings indicates the belonging of the Ca atom in these phases.

### 3.5. Thermal Analysis

**Figure 8:**
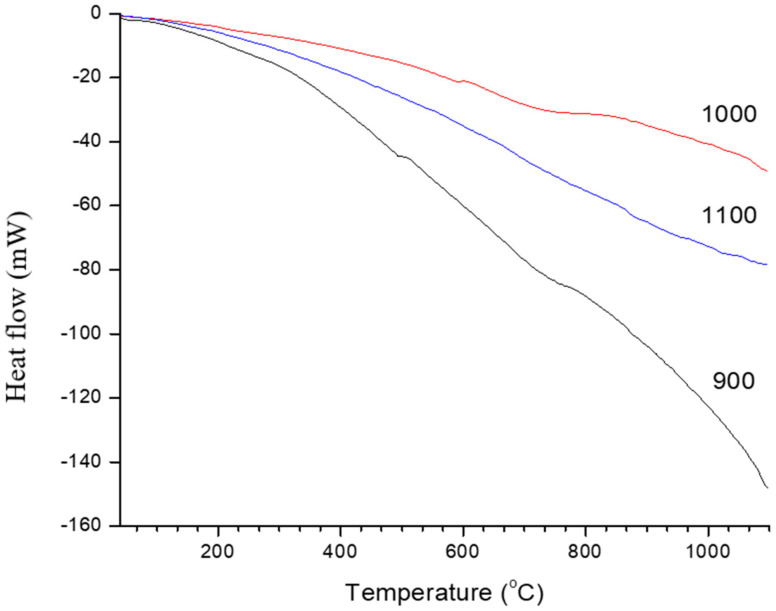
Differential scanning calorimetry (DSC) analysis of sintered kaolin-GGBS geopolymer.

Differential scanning calorimetry (DSC) analysis was performed for three samples of sintered kaolin–GGBS geopolymer, which were 900, 1000, and 1100 °C as shown in Figure 8. The 800 °C sintered samples were excluded from this analysis due to the incomplete reaction based on the microstructure and phase transformation analysis. Generally, we observed that the DSC signals increased with rising sintering temperature. Neither exothermic nor endothermic peaks were observed for 900, 1000, or 1100 °C sintered kaolin–GGBS geopolymer. The slope of the DSC curve corresponds to thermal, physical, and chemical stability of the sintered samples when exposed to 1100 °C.

The result of the sintered sample at 1000 and 1100 °C showed that it requires a temperature above 1100 °C to decompose. The stability of the sintered kaolin–GGBS geopolymer is related to the phase obtained, which is albite at all sintered samples, and other phases such as nepheline (900 °C), akermanite (1000 °C), and anorthite (1100 °C). Although the microstructure of 1000 and 1100 °C sintered samples influenced the low compressive strength, the phase obtained resulted in thermal stability.

Therefore, the sintered kaolin–GGBS geopolymer can be directly used after sintering in any application based on the phase obtained. Ye et al. suggested applications of geopolymers in the building industry where elevated temperatures may be expected, such as is in walls and floors adjacent to various heat machines [23]. Therefore, its volumetric and phase stability, and resistance to strength deterioration at high temperatures, play a significant role.

## 4. Conclusions

Generally, a fully dense ceramic material must be fabricated at very high temperatures or pressure by the hot pressing process. We found that geopolymerization is a potential initial method for producing ceramic materials at a low temperature. The sintering at 900 °C resulted in the production of sintered kaolin–GGBS geopolymer with densified microstructure and a high compressive strength of 9 MPa compared to other sintering temperatures. The low sintering temperature was contributed by Na_2_O, indicated by its formation in the albite and nepheline phase. MgO initiated the densification microstructure of the sintered geopolymer. The reaction between the chemical compositions of raw materials acted as a self-fluxing agent in reducing the temperature, which was proved from the phase transformation analysis.

## Figures and Tables

**Table 1:** Chemical composition of kaolin and ground granulated blast furnace slag (GGBS).

Chemical Composition (%)	Kaolin	GGBS
CaO	N/A	50.37
SiO_2_	54.0	30.4
Al_2_O_3_	31.7	10.5
Fe_2_O_3_	4.89	0.53
MgO	N/A	3.2
TiO_2_	1.41	0.98
K_2_O	6.05	N/A
ZrO_2_	0.10	0.05
MnO_2_	0.11	0.71
LOI	1.74	0.32

**Table 2:** Phase composition of sintered kaolin-GGBS geopolymer.

Sintering Temperature (°C)	800	900	1000	1100
Q: Quartz (SiO_2_)	77%	4%	4%	-
AL: Albite (heat treated) Na(AlSi_3_O_8_)	-	74%	95%	47%
A: Akermanite (Ca_2_Mg(Si_2_O_7_)	-	10%	1%	-
N: Nepheline (Si-rich) Na_7_ (Al_6_Si_10_O_32_)	-	13%	-	-
M: Magnetite (Fe_3_O_4_)	23%	-	-	-
AN: Anorthite (CaAl_2_Si_2_O_8_)	-	-	-	53%

## Data Availability

Data sharing is not applicable to this article.
